# Microstructure and Corrosion Behavior of Atmospheric Plasma Sprayed NiCoCrAlFe High Entropy Alloy Coating

**DOI:** 10.3390/ma15041486

**Published:** 2022-02-16

**Authors:** Kashif Mehmood, Malik Adeel Umer, Ahmed Umar Munawar, Muhammad Imran, Muhammad Shahid, Muhammad Ilyas, Rabeeka Firdous, Humaira Kousar, Muhammad Usman

**Affiliations:** 1 School of Chemical and Materials Engineering (SCME), National University of Science and Technology, Islamabad 46000, Pakistan; chkashif94@gmail.com (K.M.); ahmed.munawar@scme.nust.edu.pk (A.U.M.); mshahid@scme.nust.edu.pk (M.S.); milyas.mse14scme@student.nust.edu.pk (M.I.); rferdous.nse6scme@student.nust.edu.pk (R.F.); hkousar.mse13scme@student.nust.edu.pk (H.K.); 2 Department of Chemistry, Government College University, Lahore 54000, Pakistan; imranchemist78618@gmail.com; 3 Nust Institute of Civil Engineering (NICE), National University of Science and Technology, Islamabad 44000, Pakistan

**Keywords:** high entropy alloys, plasma spraying, corrosion, microstructure, thermal barrier coatings

## Abstract

High entropy alloys (HEAs) are multi-elemental alloy systems that exhibit a combination of exceptional mechanical and physical properties, and nowadays are validating their potential in the form of thermal sprayed coatings. In the present study, a novel synthesis method is presented to form high entropy alloy coatings. For this purpose, thermal sprayed coatings were deposited on Stainless Steel 316L substrates using atmospheric plasma spraying technique with subsequent annealing, at 1000 °C for 4 h, to assist alloy formation by thermal diffusion. The coatings in as-coated samples as well as in annealed forms were extensively studied by SEM for microstructure and cross-sectional analysis. Phase identification was performed by X-ray diffraction studies. The annealed coatings revealed a mixed BCC and FCC based HEA structure. Potentiodynamic corrosion behavior of SS316L sprayed as well as annealed coatings were also carried out in 3.5% NaCl solution and it was found that the HEA-based annealed coatings displayed the best corrosion resistance 0.83 (mpy), as compared to coated/non-annealed and SS 316 L that showed corrosion resistance of 7.60 (mpy) and 3.04 (mpy), respectively.

## 1. Introduction

High entropy alloys (HEAs) have been gaining ample attention since 1996 when Yeh et al. introduced the concept of multi-element alloy and published in 2004 [1], Later on, Cantor et al. named them ‘multi-component alloys’ in the same year [2]. The applications of HEAs in materials sectors are mainly based on their mechanical properties and durability which depend upon the existence of the second phase owing to the formation of intermetallic compounds. In the last two decades, extensive work has been going on HEAs [3] which are made of the equimolar ratio of metallic elements or concentration may vary between 5% and 35% and result in enhanced features such as mechanical strength, nanoprecipitation [4,5], extra-ordinary corrosion resistance [6], high resistivity, and magnetism [7]. These properties could be tailored further by varying and adjusting the elemental compositions that make them even more fascinating materials [6]. Thus, in addition to being a potential candidate for functional and structural materials, NiCoCrAlFe HEAs can also be applied as a surface coating to protect against corrosion and increase the durability of the material.

To date, in addition to traditional casting and forging methods, various thermal spraying techniques have been employed to coat HEAs on the substrate, firstly, high-velocity oxy-fuel (HVOF) [8,9] technique in which a specially designed torch allows the compressed flame to expand freely consuming the gases combustion, such as Hydrocarbon’s, i.e., Methane (CH_4_), Propane (C_3_H_6_), Butane (C_4_H_8_) or kerosene oil type liquid fuels. O_2_ and fuel are mixed in the combustion zone under conditions that display the correct combustion mode and pressure [10]. This process generates a very high-velocity flame which is used to propel the particles at near-supersonic speeds before making an impact on the substrate [11]. Secondly, selective laser melting (SLM) [12,13] in 3-D printing, additive or prototype manufacturing techniques [13], thirdly electro-spark deposition technique [6,14,15] Fourthly, laser cladding [16,17] but all these techniques have a shortcoming in terms of low deposition rate, higher cost, and fuel energy requirement.

The atmospheric plasma spraying technique [15,18,19] has emerged as a promising method to spray HEAs on the substrate. In this spraying method, feedstock (powder, wire, liquid, or suspension form) is deposited on the substrate after heating at a temperature of around 10,000 K to 15,000 K [20,21] Semi-molten or molten drops flatten, promptly solidify and form a solid deposit. Generally, the deposits stay adherent to the substrate as a coating [22,23]. The HEAs with Nickel elements have the prodigious potential for mechanical application [24]. Although, corrosion resistance studies are unavoidable for their prospective application in some mediums especially in saline medium (3.5% NaCl) as the corrosion behavior of HEAs is comparatively unfamiliar. The most commonly used engineering material is stainless steel, its erosion behavior in NaCl (3.5%) has gained trivial interest over a long period. It is documented that several alterations have been employed to stainless steel to avoid corrosion and effect of various factors also have been studied including sensitization, alloying, annealing, coatings, and selective laser melting [25]. So, there is a need to investigate and compare the corrosion behavior of HEAs with commonly available ferrous alloys such as Steel 316L [26]. Recently, the corrosion behavior of various HEAs using plasma spray method has been reported similar to (CoCrFeNi)_95_Nb_95_ coating on Q235 steel substrate was carried out using plasma spraying having a thickness of 500 µm [27]. Similarly, the corrosion behavior of NiCoCrAlFe-M HEACs (HEA composites) and AlCoCrFeCu-X_0.5_ has also been reported [17,28,29], all of these HEAs were coated using laser surface alloying techniques. 

In the current study, NiCoCrAlFe HEA-based coatings were thermally sprayed onto the surface of steel 316L using the plasma spray method. The resultant HEA coatings were characterized for their morphology and structure evolution as a function of annealing using advanced characterization techniques including SEM and XRD. Furthermore, the corrosion behavior of NiCoCrAlFe HEAs was tested for steel in 3.5% NaCl media in an as deposited and annealed state.

## 2. Materials and Methods

### 2.1. Chemicals

The elemental powders of Nickel, Cobalt, Chromium, Aluminum, Iron with purity >99.5% were purchased from GREEN EARTH CHEM West Nanjing Road, Shanghai, China, and Ball Milling/Blending was carried out for homogenous mixing of elemental powders.

### 2.2. Sample Preparation and Pre-Heat Treatments

All the elemental powders used, having purity >99.5% were placed in a polypropylene jar in equal-atomic ratios in a Glove Box to avoid oxidation. After that stainless steel balls of Ø10 mm and Ø8 mm were inserted into the jar and Argon was filled in the jar to keep the atmosphere inert during ball milling. The speed of the Ball mill apparatus was set to 110 rpm and the ball to powder weight ratio (mass ratio) was 10:1. Mixing of elemental powders was carried out for 10 h in a 2-roll ball milling apparatus Wise mill (BML 5) Labortechnik GmbH Witeg Germany. Stainless Steel 316L substrates were used as substrate material for coatings. The steel may contain grease or oil and do not have fine surface properties. For coating, the steel underwent various steps including surface cleaning, shaping, and surface activation. Shaping was carried out using a diamond cutter and also Electrical discharge machining (EDM) also known as Spark Machining. The substrates were cut into 1-inch × 1-inch dimensions from a blank plate. After that cleaning was carried out by dipping the substrates in acetone (CH_3_COCH_3_) to remove impurities and dirt. To increase the surface roughness of our shaped substrates grit blasting was performed at a pressure of 0.35 MPa followed by air drying. Alumina (Al_2_O_3_) particles have a particle size of 40~60 µm. Air drying was carried out to remove any debris or dust on the surface. The pressure for air drying was kept slightly lower than the pressure used for grit blasting to avoid any surface damage.

### 2.3. Atmospheric Plasma Spraying Coating of Samples

Pre-heating of the processed HEA powder was carried out by placing it in the oven for 2 h at 25–150 °C, to remove any moisture present. The substrates were pre-heated with the flame of the spraying gun before the powder feed start. Plasma sprayed samples were manufactured by conventional Atmospheric Plasma Spraying system (Metco 9MC, Sulzer Metco Inc., Westbury, NY, USA) using a stand-off distance of 250 mm. Argon (Ar) gas was employed as the primary plasma operating gas and hydrogen (H_2_) was used as the secondary gas to escalate the whole plasma jet temperature. The feedstock ball milled powder was conveyed by a separate argon source and inoculated superficially into the spawned plasma jet at an angle of 90° concerning the anode axis. After that plasma torch assembly was mounted onto a robotic arm (YR-SK16-J00 Motoman, Yaskawa Electric Corp, Fukuoka, Japan) to navigate across the substrate holders for coating. All the parameters of the plasma spraying operation are enlisted in Table 1.

### 2.4. Annealing of Coatings

After the fabrication of APS coatings, the annealing was carried out in a Tube Furnace (Protherm PZF 12/50/700) for 4 h at 1000 °C in an inert atmosphere for homogenization and elemental diffusion of the thermally sprayed APS coatings.

### 2.5. Characterization and Corrosion Studies of HEA

Microstructure and cross-section analysis of the coatings in as-coated condition were performed on a Leo Gemini Analytical SEM (ZEISS, Oberkochen, Germany) and in annealed conditions using VEGA 3 TESCAN SEM by varying the magnification. Point Elemental Analysis was carried out at different phases of the annealed coatings on behalf of color contrast using an energy dispersive X-rays spectrometer (Oxford Instrument, INCAxcat detector, Abingdon, UK,) attached with SEM. XRD analysis for phase detection was carried out using a STOE Panalytical X-ray diffractometer (XRD, STOE Inc., Darmstadt, Germany) with Cu-K_α_ radiation using a step size of 0.02° and time 20 s. Micro Vickers hardness of the SS 316L substrate and the coatings was performed using a micro tester (Duramin, Struers, Denmark) under a load of 100 gm and a dwell time 15 s. Surface roughness of substrate as well as plasma-sprayed coatings and annealed coatings was measured using a SE700 Roughness Tester (Kosaka Laboratory, Tokyo, Japan).

The electrochemical corrosion testing was evaluated using Potentiostat/Galvanostat interface 1010E (Gamry Instruments, Warminster, PA, USA) in 3.5% NaCl solution. Wires were spot welded at back of each sample to make connections and then mounted in epoxy having exposed area of 1 cm^2^ to be used as working electrode. Ag/AgCl in a saturated solution of KCl was used as reference electrode. Parallel graphite rod was engaged as the counter electrode. The potentiodynamic polarization test was executed at a scan rate of 2 mV/s from an initial potential of −0.25 V to a final potential value of 1.25 V against the OCP. The testing was carried out four times to confirm repeatability.

## 3. Results and Discussion

### 3.1. Microstructural Analysis

**Figure 1:**
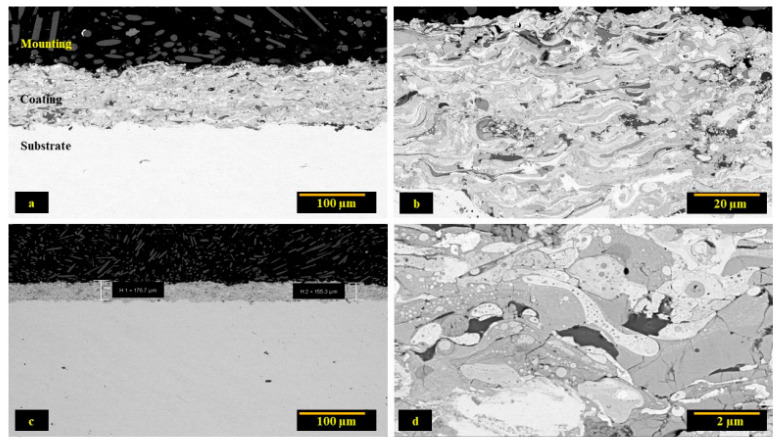
SEM micrographs of As-sprayed Coatings at different magnifications (**a**) Dense coating (**b**) Lamellar microstructure of coating (**c**) Coating thickness (**d**) Microstructure at higher magnification.

The SEM images (Figure 1) show that the coatings exhibit a splat morphology containing dark and light phases. This can be attributed to the melting of particles owing to the high temperature of the plasma torch while few particles that experience lower temperatures can be seen to exist in a partially molten state also adhere to the substrate. The SEM image also revealed that the resultant microstructure was homogenized and the coatings have well adhered to the substrate with little porosity. The coatings have an average thickness of 150~180 µm.

**Figure 2:**
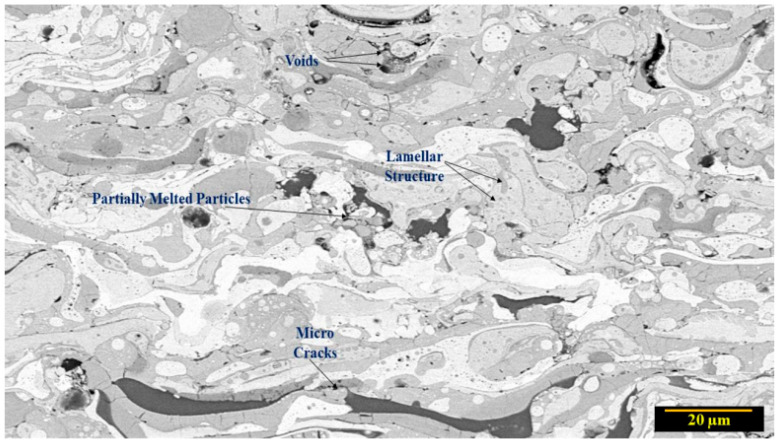
Enlarged microstructural view of the as-sprayed coatings.

An enlarged microstructural view of the as-sprayed coatings is shown in (Figure 2), it can be seen that few oxide splats (light grey), voids, micro-cracks, partially molten particles, and the lamellar structure were present same as reported earlier by Ang et al. [30], but their ratio to the homogenized structure is negligible.

**Figure 3:**
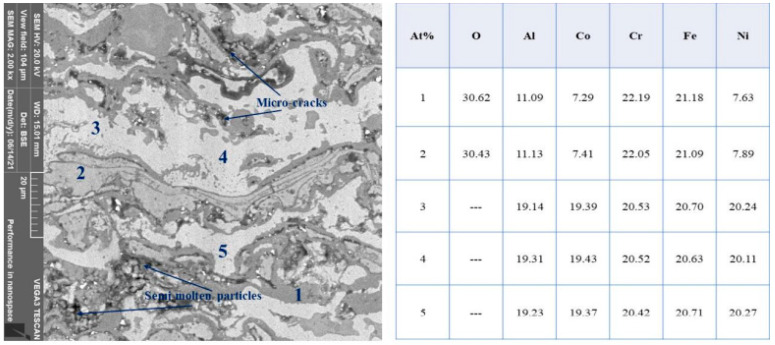
Microstructure of Annealed Coating (**left**) and EDS at different Points (**right**).

Annealing treatment for 4 h at 1000 °C in an inert atmosphere was performed on the plasma sprayed coatings. The microstructure of the annealed coatings revealed a lamellar structure similar to the as-coated form, but a major change was observed after annealing treatment of the spayed substrates such as alloy fraction increased as shown in (Figure 3). Three types of phases were detected based on color contrast, i.e., dark gray phase, light gray phase, and white phase. As reported earlier by Meghwal et al. [31], the dark grey phase consists mainly of spinels, and other oxides solidified after plasma spraying while the white phase is composed of a solid solution of the metal powders used for coating. After annealing, it can be seen that the amount of white phase fraction increased, evident in Figure 3. EDS analysis of the annealed samples performed at various points mentioned as points 1 and 2, corresponding to the grey phase, reveals large atomic fractions of oxygen suggesting the formation of oxide(s). However, EDS analysis of points 3, 4, and 5, all pertaining to the light phase shows no trace of oxygen. Furthermore, all constituent elements used were present in equal ratios in these regions indicating the formation of an equiatomic solid solution.

Alongside the high entropy solid solution formation, oxide stringers which appeared as light and dark grey phases were seen to exist, which is inherent owing to the high temperatures and oxidizing conditions of plasma flame. The annealing at 1000 °C for the stipulated time proved to be sufficient for the diffusion of atoms to form HEA which was not achieved in coated samples. The presence of an inert atmosphere with Argon gas restricted any oxide formation during annealing.

### 3.2. Phase Analysis

**Figure 4:**
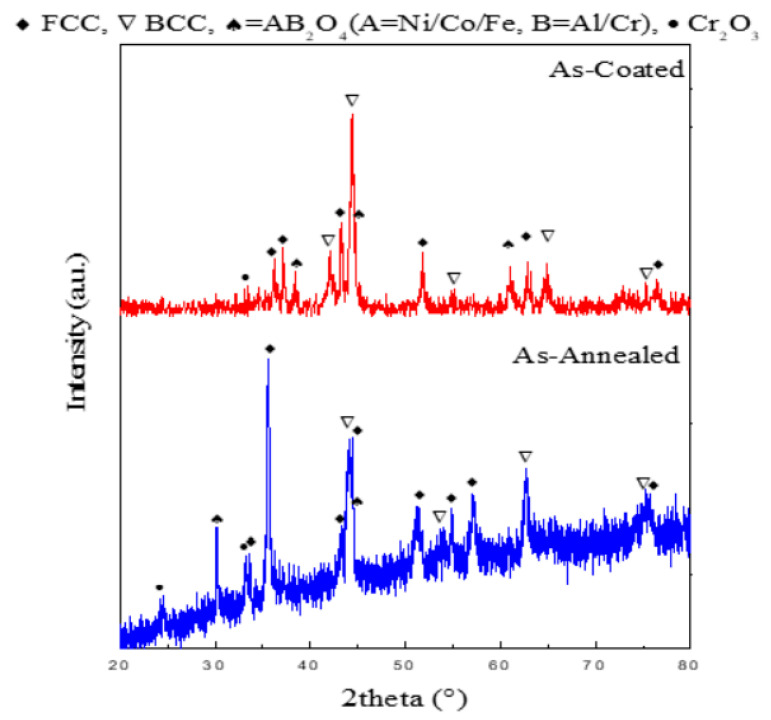
XRD analysis of the coatings in as-coated and as-annealed conditions.

XRD analysis was performed for the crystal structure identification and phase analysis of the high entropy alloy coatings. The analysis was performed in as-coated as well as in annealed states of coatings in order to attain a complete picture and understanding of the phase evolution taking place. As shown in (Figure 4), Atmospheric Plasma sprayed substrates in the as coated conditions revealed a multitude of BCC and FCC phases. It can be seen that as-sprayed coatings mainly consisted of pure metals having BCC and FCC crystal structures, except some quantities of Fe-Cr alloy, FeO, and spinel were detected. Due to the high plasma jet temperatures, in-flight oxidation occurs in the APS process leading to peaks of mixed spinel oxides, marked as ♠, AB_2_O_4_ (A = Ni/Co/Fe, B = Al/Cr) were also detected [32].

All the diffraction peaks correspond well with the already published work [33]. Major peaks representing the BCC and FCC are found to be present. Some of the diffraction peaks are slightly shifted in comparison to already published work on this alloy system because of the difference in amounts of solid solution formation due to a difference in the atomic radii of Ni, Fe, Cr, Al, and Co.

After an annealing treatment at 1000 °C, the content of Cr-O increased in the coatings while the presence of metals in their pure form was minimized, similarly found by Lin et al. [33]. In the case of annealing treatment, the following changes were observed; an increase in intensity at 2θ: 37.16°, the peak at 2θ: 38° for spinel changed to FCC with an increased intensity which may be attributed to solid solution FCC lattices, and peak broadening took place at 2θ: 44.16° which indicates the presence of fine structure and appeared as FCC, BCC and spinel after annealing. Due to the effects of heat treatment, elemental diffusion took place to a larger extent sufficiently which enhanced the entropy of our system significantly and restricted the formation of the intermetallic compounds which are the ordered phases. The increase in the entropy of the system reduced the free energy of the alloy system thus, making stable solid solutions [31,33]. It was revealed that BCC and FCC exist as the major phases present in the coating structure.

### 3.3. Micro Vickers Hardness

**Figure 5:**
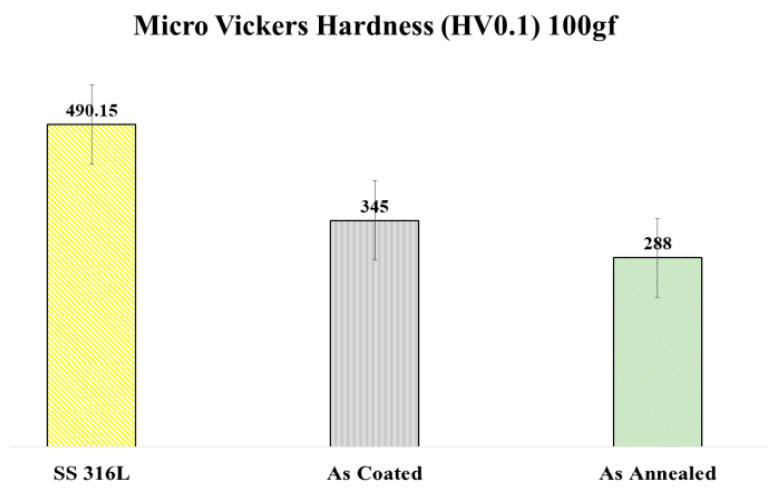
Micro Hardness Values of SS 316L, APS Coating and Annealed Coating.

The hardness can be referred to as the capability of a material to resist localized plastic deformation. Five tests were performed for each sample and readings were recorded as average values with standard deviation. Munitz et al. [25] reported that the microhardness value in as-cast condition is higher than after various heat treatments for AlCoCrFeNi alloy. The hardness values after heat treatment for different ranges of temperature leads to phase transformations [25]. The possible reason for higher hardness in as-coated coatings is the rapid solidification of the melted particles at the substrate surface which is similar to quenching, due to which the dislocations and discontinuities become embedded in the microstructure in a staggered manner which induces stresses in the microstructure. After annealing of sprayed coatings at 1000 °C for 4 h microhardness was reduced to some extent due to reduction of dislocations in the microstructure [34,35]. Annealing of coatings favored the diffusion of elements with each other and HEA formation. Alloy fractions have lower microhardness values than the oxide phases present in the lamellar structure as reported by Meghwal et al. [31]. Results for the microhardness value of Bare SS 316L substrate, APS coating and annealed coatings are also shown graphically below in (Figure 5).

### 3.4. Roughness Testing

**Figure 6:**
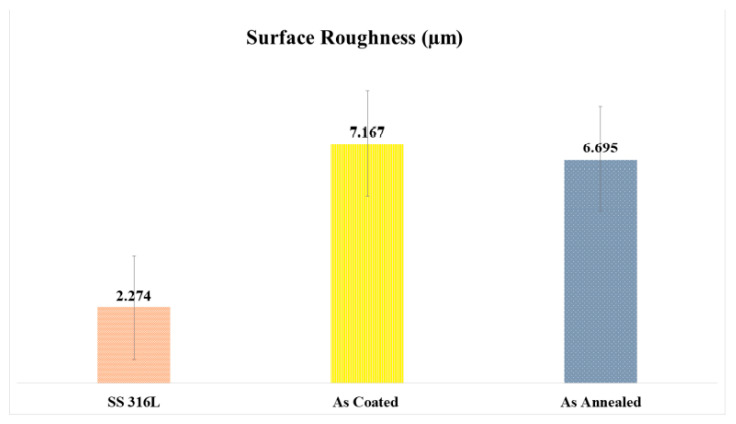
Surface Roughness (µm).

The roughness of the thermal sprayed coatings can be adjusted by optimizing the parameters while spraying that include stand-off distance, power, voltage, and feedstock morphology as reported by Anupam et al. [36]. Li, Zuhao, et al. reported that the roughness of the alloy improved after the heat treatment [37]. In the case of plasma-sprayed samples, the roughness values were found to be higher than that of annealed coatings. Different particles experience different heating temperatures during flight and projection towards substrate thus forming splats on the substrate surface upon complete or partial melting. These partially melted particles forming splats upon cooling give rise to higher values of roughness. While in the case of annealing treatment of coatings the roughness values were found to be lowered, this is consistent with the diffusion process as the temperature applied in annealing favored the alloy formation and grain refinement which resulted in a fine microstructure and lower roughness values. The results are also summarized below in the graphical form shown in (Figure 6).

### 3.5. Potentiodynamic/Corrosion Testing

**Figure 7:**
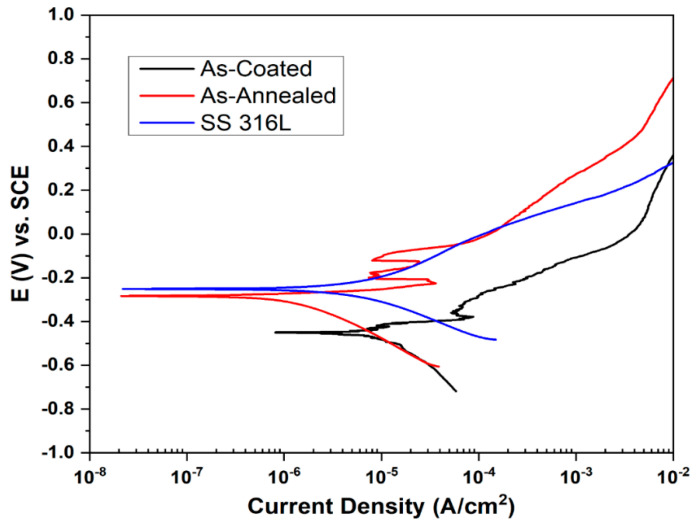
Curves depicting Potentiodynamic polarization/Corrosion performance of SS 316L, Coated and Annealed Sample in 3.5% NaCl.

The Potentiodynamic polarization behavior of bare stainless steel 316L, HEA coated, and annealed plasma sprayed coatings in 3.5% NaCl are shown in the (Figure 7).

Critical information such as corrosion potential (E_corr_), current density (I_corr_) can be inferred from potentiodynamic polarization curves that play vital role in determining corrosion behavior of a material. It can be seen that active to passive transition zone can be seen in coated as well as in annealed coating while substrate was unable to develop it. This can be attributed to delay in the formation of a protective film on the substrate surface as reported by Shi et al. [38]. In case of stainless steel, the results revealed that with current density of 7.32 µA/cm^2^, the surface of bare stainless steel is affected by 3.5% NaCl solution and becomes corroded. This can be attributed as stabilization of pitting. The E_corr_ value was calculated to be −0.245 V with anodic slope of 0.215 and a cathodic slope of −0.167. In case of as coated stainless steel substrate, the results revealed that the current density I_corr_ was 18.31 µA/cm^2^ which is significantly higher than that of the stainless steel as well as the APS sprayed annealed coating. This reflects that coating was not dense enough, contained elements in their free form as detected by XRD analysis shown in Figure 4 and were not diffused enough to form complete alloy fractions and it had potential corrosion sites exposed which were more prone to the chlorides attack on the surface. The driving force for the fabrication of corrosion-resistant NiCoCrAlFe based HEA coatings lies in the ease of formation of a protective film that is based on principal alloying elements Ni, Al, and Cr. The value of current density wasn’t reduced by the prepared coating, as HEA wasn’t formed well enough at this stage and elements were present in the coatings thus showing the lowest corrosion resistance and highest value of current density. The corresponding values of E_corr_ were −0.454 V with anodic and cathodic slope values 0.072 and −0.169, respectively. In the case of annealed coating, the large passive region and lower values of I_Corr_ results revealed that the current density was 1.99 µA/cm^2^ which is significantly less than that bare stainless steel 316L and HEA coating by APS on it. The corrosion resistance of annealed coating may be attributed to the formation of an oxide layer on the substrate which results in lessening the dissolution corrosion susceptible phase by impeding electrolyte. This oxide film inhibits the electrolyte attack and diminishes the suspension of the corrosion-prone phases [39]. The curve directly transformed from the Tafel region to the passive region and this can be ascribed to the prompt formation of a protective film on the surface of coating at corrosion potential which decreased the current density. A similar type of polarization curve was reported by Shi et al. [40,41] while analyzing the electrochemical performance of cast Al_x_CoCrFeNi HEAs in 3.5 wt % NaCl solution. This phenomenon increased its corrosion resistance performance as compared to bare SS 316L substrate as well as a sample in as-coated condition. The corresponding value of E_corr_ was −0.281 with anodic and cathodic slope 0.052 and −0.232, respectively. All the electrochemical values are given in Table 2.

## 4. Conclusions

The following conclusions can be drawn from the present studies:

1. APS sprayed coatings consist of a multitude of phases scattered, i.e., bcc and fcc in the lamellar structure of the prepared coatings.
2. High Entropy Alloy NiCoCrAlFe has been successfully formed upon annealing of the thermal sprayed coatings in the inert atmosphere.
3. It was found that HEA annealed coatings have slightly higher corrosion resistance, both general and localized than SS 316L substrate as well as the APS sprayed coatings. This makes them a potential candidate for use as protective coatings.
4. Annealed coatings showed the best Corrosion resistance against chloride attacks among the substrate and sprayed coatings while examining their Potentiodynamic behavior in 3.5% NaCl Solution.

## Figures and Tables

**Table 1:** Plasma Spraying Operating Parameters.

Parameters	Value
Arc voltage (V)	70
Arc current (A)	600
Powder Feed rate (g/min)	30
Primary Gas Flow (Ar/slpm)	40
Secondary Gas Flow (H_2_/slpm)	2
Powder Feed Gas Flow (Ar/slpm)	5
Stand-Off Distance (mm)	250
Traverse speed of torch (mm/s)	200
Angle	90°
Nozzle Type	9 MB
Controller	9 MC

**Table 2:** Electrochemical data of Stainless Steel 316L, Coated Sample and Annealed Sample acquired after Corrosion Behaviour Testing in 3.5% NaCl solution.

Sample	E_corr_ (mV_SCE_)	Ba (V) Anodic Slope	Bc (V) Cathodic Slope	I_Corr_ (µA/cm^2^)	Corrosion Rate CR (mpy)
SS 316L	−0.245	0.215	−0.167	7.32	3.04
As-Coated	−0.454	0.047	−0.271	18.31	7.60
As-Annealed	−0.281	0.052	−0.232	1.99	0.83

## Data Availability

The data presented in this study are available on request from the corresponding author.

## References

[1] Yeh J.-W., Chen S.K., Lin S.-J., Gan J.-Y., Chin T.-S., Shun T.-T., Tsau C.-H., Chang S.-Y. (2004). Nanostructured High-Entropy Alloys with Multiple Principal Elements: Novel Alloy Design Concepts and Outcomes. Adv. Eng. Mater..

[2] Cantor B., Chang I.T.H., Knight P., Vincent A.J.B. (2004). Microstructural development in equiatomic multicomponent alloys. Mater. Sci. Eng. A.

[3] Yu J., Lin X., Wang J., Chen J., Huang W. (2009). First-principles study of the relaxation and energy of bcc-Fe, fcc-Fe and AISI-304 stainless steel surfaces. Appl. Surf. Sci..

[4] Li Z., Zhao S., Ritchie R.O., Meyers M.A. (2019). Mechanical properties of high-entropy alloys with emphasis on face-centered cubic alloys. Prog. Mater. Sci..

[5] Diao H., Xie X., Sun F., Dahmen K.A., Liaw P.K. (2016). Mechanical Properties of High-Entropy Alloys. High-Entropy Alloy..

[6] Zhang Y., Zuo T.T., Tang Z., Gao M.C., Dahmen K.A., Liaw P.K., Lu Z.P. (2014). Microstructures and properties of high-entropy alloys. Prog. Mater. Sci..

[7] Qiu X.-W., Zhang Y.-P., He L., Liu C.-G. (2013). Microstructure and corrosion resistance of AlCrFeCuCo high entropy alloy. J. Alloy. Compd..

[8] Kandeva M., Zadorozhnaya E., Kalitchin Z.H., Svoboda P. (2018). Tribological Studies of High Velocity Oxy-Fuel (HVOF) Superalloy Coatings. J. Balk. Tribol..

[9] Sidhu T., Prakash S., Agrawal R. (2006). Hot corrosion behaviour of HVOF-sprayed NiCrBSi coatings on Ni- and Fe-based superalloys in Na_2_SO_4_–60% V2O5 environment at 900 °C. Acta Mater..

[10] Mora-García A., Ruiz-Luna H., Mosbacher M., Popp R., Schulz U., Glatzel U., Muñoz-Saldaña J. (2018). Microstructural analysis of Ta-containing NiCoCrAlY bond coats deposited by HVOF on different Ni-based superalloys. Surf. Coat. Technol..

[11] Srivastava M., Jadhav M., Chakradhar R.P.S., Muniprakash M., Singh S. (2019). Synthesis and properties of high velocity oxy-fuel sprayed FeCoCrNi2Al high entropy alloy coating. Surf. Coat. Technol..

[12] Yap C.Y., Chua C.K., Dong Z.L., Liu Z.H., Zhang D.Q., Loh L.E., Sing S.L. (2015). Review of selective laser melting: Materials and applications. Appl. Phys. Rev..

[13] Lapin J., Pelachová T., Dománková M.J.I. (2018). Long-term creep behaviour of cast TiAl-Ta alloy. Intermetallics.

[14] Ruijun W., YiYu Q., Jun L. (2004). Interface behavior study of WC92–Co8 coating produced by electrospark deposition. Appl. Surf. Sci..

[15] Kirik G., Gaponova O.P., Tarelnyk V., Myslyvchenko O., Antoszewski B. (2018). Quality Analysis of Aluminized Surface Layers Produced by Electrospark Deposition. Powder Met. Met. Ceram..

[16] Wu C.L., Zhang S., Zhang C.H., Chen J., Dong S.Y. (2017). Phase evolution characteristics and corrosion behavior of FeCoCrAlCu-X_0.5_ coatings on cp Cu by laser high-entropy alloying. Opt. Laser Technol..

[17] Jin G., Cai Z., Guan Y., Cui X., Liu Z., Li Y., Dong M., Zhang D. (2018). High temperature wear performance of laser-cladded FeNiCoAlCu high-entropy alloy coating. Appl. Surf. Sci..

[18] Gill B., Tucker R.C. (1986). Plasma spray coating processes. J. Mater. Sci. Technol..

[19] Fauchais P., Vardelle M., Bianchi L. (1996). Plasma spray: Study of the coating generation. Ceram. Int..

[20] Löbel M., Lindner T., Kohrt C., Lampke T. Processing of AlCoCrFeNiTi high entropy alloy by atmospheric plasma spraying. Proceedings of the IOP Conference Series: Materials Science and Engineering.

[21] Paleu C., Munteanu C., Istrate B., Bhaumik S., Vizureanu P., Bălţatu M., Paleu V. (2020). Microstructural Analysis and Tribological Behavior of AMDRY 1371 (Mo–NiCrFeBSiC) Atmospheric Plasma Spray Deposited Thin Coatings. Coatings.

[22] Xiao J.-K., Tan H., Wu Y.-Q., Chen J., Zhang C. (2020). Microstructure and wear behavior of FeCoNiCrMn high entropy alloy coating deposited by plasma spraying. Surf. Coat. Technol..

[23] Istrate B., Rau J.V., Munteanu C., Antoniac I.V., Saceleanu V. (2020). Properties and in vitro assessment of ZrO2-based coatings obtained by atmospheric plasma jet spraying on biodegradable Mg-Ca and Mg-Ca-Zr alloys. Ceram. Int..

[24] Ríos M.L., Perdomo P.P.S., Voiculescu I., Geanta V., Crăciun V., Boerasu I., Rosca J.C.M. (2020). Effects of nickel content on the microstructure, microhardness and corrosion behavior of high-entropy AlCoCrFeNix alloys. Sci. Rep..

[25] Munitz A., Salhov S., Hayun S., Frage N. (2016). Heat treatment impacts the micro-structure and mechanical properties of AlCoCrFeNi high entropy alloy. J. Alloy. Compd..

[26] Zheng Z.Y., Li X.C., Zhang C., Li J.C. (2015). Microstructure and corrosion behaviour of FeCoNiCuSnx high entropy alloys. Mater. Sci. Technol..

[27] Wang W., Qi W., Xie L., Yang X., Li J., Zhang Y. (2019). Microstructure and Corrosion Behavior of (CoCrFeNi)95Nb5 High-Entropy Alloy Coating Fabricated by Plasma Spraying. Materials.

[28] Ye Q., Feng K., Li Z., Lu F., Li R., Huang J., Wu Y. (2017). Microstructure and corrosion properties of CrMnFeCoNi high entropy alloy coating. Appl. Surf. Sci..

[29] Jin B., Zhang N., Guan S., Zhang Y., Li D. (2018). Microstructure and properties of laser re-melting FeCoCrNiAl0.5Six high-entropy alloy coatings. Surf. Coat. Technol..

[30] Ang A.S.M., Berndt C.C., Sesso M.L., Anupam A., Praveen S., Kottada R.S., Murty B.S. (2015). Plasma-Sprayed High Entropy Alloys: Microstructure and Properties of AlCoCrFeNi and MnCoCrFeNi. Metall. Mater. Trans. A.

[31] Meghwal A., Anupam A., Luzin V., Schulz C., Hall C., Murty B., Kottada R.S., Berndt C.C., Ang A.S.M. (2021). Multiscale mechanical performance and corrosion behaviour of plasma sprayed AlCoCrFeNi high-entropy alloy coatings. J. Alloy. Compd..

[32] Imran M., Saeed Z., Pervaiz M., Mehmood K., Ejaz R., Younas U., Nadeem H.A., Hussain S. (2021). Enhanced visible light photocatalytic activity of TiO2 co-doped with Fe, Co, and S for degradation of Cango red. Spectrochim. Acta Part A Mol. Biomol. Spectrosc..

[33] Lin D.-Y., Zhang N.-N., He B., Jin B.-Q., Zhang Y., Li D.-Y., Dong F.-Y. (2017). Influence of laser re-melting and vacuum heat treatment on plasma-sprayed FeCoCrNiAl alloy coatings. J. Iron Steel Res. Int..

[34] Szala M., Winiarski G., Wójcik Ł., Bulzak T. (2020). Effect of Annealing Time and Temperature Parameters on the Microstructure, Hardness, and Strain-Hardening Coefficients of 42CrMo4 Steel. Materials.

[35] Godoy C., Souza E.A., Lima M.M., Batista J.C.A. (2002). Correlation between residual stresses and adhesion of plasma sprayed coatings: Effects of a post-annealing treatment. Thin Solid Films.

[36] Anupam A., Kottada R.S., Kashyap S., Meghwal A., Murty B., Berndt C., Ang A.S. (2020). Understanding the microstructural evolution of high entropy alloy coatings manufactured by atmospheric plasma spray processing. Appl. Surf. Sci..

[37] Li Z., Liu C., Wang B., Wang C., Wang Z., Yang F., Gao C., Liu H., Qin Y., Wang J. (2018). Heat treatment effect on the mechanical properties, roughness and bone ingrowth capacity of 3D printing porous titanium alloy. RSC Adv..

[38] Li P., Wang A., Liu C. (2017). A ductile high entropy alloy with attractive magnetic properties. J. Alloy. Compd..

[39] Jiang Y., Li J., Juan Y., Lu Z., Jia W. (2019). Evolution in microstructure and corrosion behavior of AlCoCrxFeNi high-entropy alloy coatings fabricated by laser cladding. J. Alloy. Compd..

[40] Shi Y., Collins L., Feng R., Zhang C., Balke N., Liaw P.K., Yang B. (2018). Homogenization of AlxCoCrFeNi high-entropy alloys with improved corrosion resistance. Corros. Sci..

[41] Shi Y., Yang B., Xie X., Brechtl J., Dahmen K.A., Liaw P.K. (2017). Corrosion of Al xCoCrFeNi high-entropy alloys: Al-content and potential scan-rate dependent pitting behavior. Corros. Sci..

